# Bleeding skin lesions in gestating sows of a piglet producing farm in Austria

**DOI:** 10.1186/s40813-023-00348-4

**Published:** 2023-11-14

**Authors:** Lukas Schwarz, Flora Hamar, Tanja Bernreiter-Hofer, Igor Loncaric, Mirjam Arnold, Thomas Voglmayr, Andrea Ladinig

**Affiliations:** 1 University Clinic for Swine, Department for Farm Animals and Veterinary Public Health, University of Veterinary Medicine Vienna, Vienna, Austria; 2 Institute of Microbiology, Department of Pathobiology, University of Veterinary Medicine Vienna, Vienna, Austria; 3 Clinic for Swine, Department for Clinical Veterinary Medicine, Vetsuisse Faculty, University of Bern, Bern, Switzerland; 4 Traunkreis Vet Clinic, Grossendorf, Austria; 5 Present Address: Tierarztpraxis an der Nordbahn, Strasshof, Austria; 6 Present Address: Institute for Animal Health I, Bavarian Health and Food Safety Authority, Oberschleißheim, Germany

**Keywords:** *Stomoxys calcitrans*, Stable fly, Fly control

## Abstract

**Background:**

*Stomoxys calcitrans*, the stable fly, occurs in pig producing countries worldwide. While in cattle the impact of this blood sucking insect is quite well described, its role in pig production is poorly investigated. Here we describe a case of a massive stable fly overpopulation in the gestation unit of a piglet producing farm in Austria that resulted in bleeding skin lesions in bitten sows.

**Case Presentation:**

In October 2021, the responsible herd veterinarian of the case farm reported of sows in the gestation area presenting with bloody crusts on the whole skin surface of the body and of bleeding skin lesions. 33/55 sows were affected by moderate to severe skin lesions. Reproductive performance decreased during the time of massive stable fly overpopulation. Sows in the gestation unit showed defensive behaviour and at a certain time point resigned and accepted being bitten by stable flies. After controlling the fly population, reproductive performance improved and even exceeded the performance before the massive overgrowth of the stable fly population.

**Conclusions:**

Stable flies are a serious harm to pigs and should be kept in mind for improved animal health and welfare. Knowledge about the determination of *Stomoxys calcitrans* and early recognition of an increasing stable fly population in pig farming systems followed by proper insect control measures have to be performed to reduce losses caused by this harming insect.

**Supplementary Information:**

The online version contains supplementary material available at 10.1186/s40813-023-00348-4.

## Background

The stable fly, *Stomoxys calcitrans*, is present in pig producing countries all over the world. Its relevance in pig production is hardly understood. The stable fly can have an impact on pig health and welfare. Restlessness, pain due to biting, stress, loss of blood, reduced feed intake and local skin inflammation after biting are direct influences affecting the bitten host [1, 2]. The role of the stable fly as a real biohazard in pig producing systems is poorly investigated. However, historic literature described large outbreaks of stable flies in the United States from 1894 to 1912 that had not only impact on animals and humans, but rather an economic impact due to production losses in dairy cows [3].The biology of this blood feeding insect is quite different to the commonly known house fly and reproduction is linked to decomposing organic matter, such as plant materials [3–6]. Stable flies reproduce in temperature ranges from 15 to 30 °C and reach their adult stage as imago under optimal conditions after 13 days [4]. Female stable flies deposit on average 292 eggs over a two-week laying period [4], consequently large populations may develop in a short time period. The effect of stable fly bites on host animals is avoiding behaviour to prohibit fly bites, consequently it disturbs animals in their natural behavioural needs, such as resting and feeding [3]. Under circumstances of large stable fly populations animals may give up fighting against the fly bites and stable flies can proceed in blood sucking, indicating the importance of these pest insect in food animal production systems [5]. Mechanical injuries in pigs due to the bites are often observed on soft skin parts such as behind the ears [5].

However, the stable fly may also act as a vector transmitting pathogens. Besides important infectious agents, such as African swine fever virus [7], a recent study investigating stable flies in piglet producing farms in Austria assumed them as possible vectors for porcine circovirus 2 and hemotropic mycoplasma species. In this study, it was also shown that a total of 69 different microorganisms could be detected on the surface of stable flies [8].

In this case report we describe a severe outbreak of *Stomoxys calcitrans* resulting in a stable fly overgrowth in the gestation area of a piglet producing farm in Austria with the consequence of severe bleeding skin lesions, restlessness and reduced reproductive performance of gestating sows.

## Case presentation

### Anamnesis

In October 2021, the responsible herd veterinarian of an Austrian piglet producing farm contacted the University Clinic for Swine, University of Veterinary Medicine Vienna, Austria and reported of sows in the gestation area presenting with bloody crusts on the whole skin surface of the body and of bleeding skin lesions. This sarcoptic mange free farm produced piglets in a three-week batch farrowing interval with 105 cross-bred sows (Large White x Landrace). Gilts were obtained from one sarcoptic mange negative gilt supplying farm and were kept in a separate isolation unit before being integrated in the sow herd. Prior to integration gilts were vaccinated against parvovirosis and erysipelas (Parvoruvac®, Merial SAS, Lyon, France) and treated with ivermectine (Ivomec 10 mg/ml, Boehringer Ingelheim Animal Health France SCS, Lyon, France) to prevent introduction of sarcoptic mange and round worms. Sows were dewormed twice a year alternately with fenbendazole (Panacur 4%, Intervet, Vienna, Austria) and flubendazole (Flubenol 50 mg/g, Elanco, Cuxhaven, Germany).

The farmer reported that in July 2021 a general cleaning of the gestation area was performed, when the sows were removed in two steps for cleaning using a high-pressure cold-water washer. Approximately three to four days thereafter a fast growing fly population could be observed in the gestation area. At that time the fly species was not determined, since neither the farmer nor the herd attending veterinarian were able to distinguish stable flies from house flies. The farmer used insecticides in August to control the fly population. From mid-October 2021 onwards, the fly population increased again since liquid manure from the nursery units was directed via a direct connection pipe to the slurry channel of the gestation area to make liquid manure more fluid and to break the crust of the manure cover. However, at that time the farmer and the herd attending veterinarian observed sows suffering from skin lesions that were either covered by bloody crusts or were bleeding. Additionally, the farmer reported of increased return-to-oestrus rates at that time, which were thought being related to a broken climate chamber for semen storage. At this time point the University Clinic for Swine got involved in the diagnostic work up.

### Herd inspection and clinical examination of sows

**Fig. 1:**
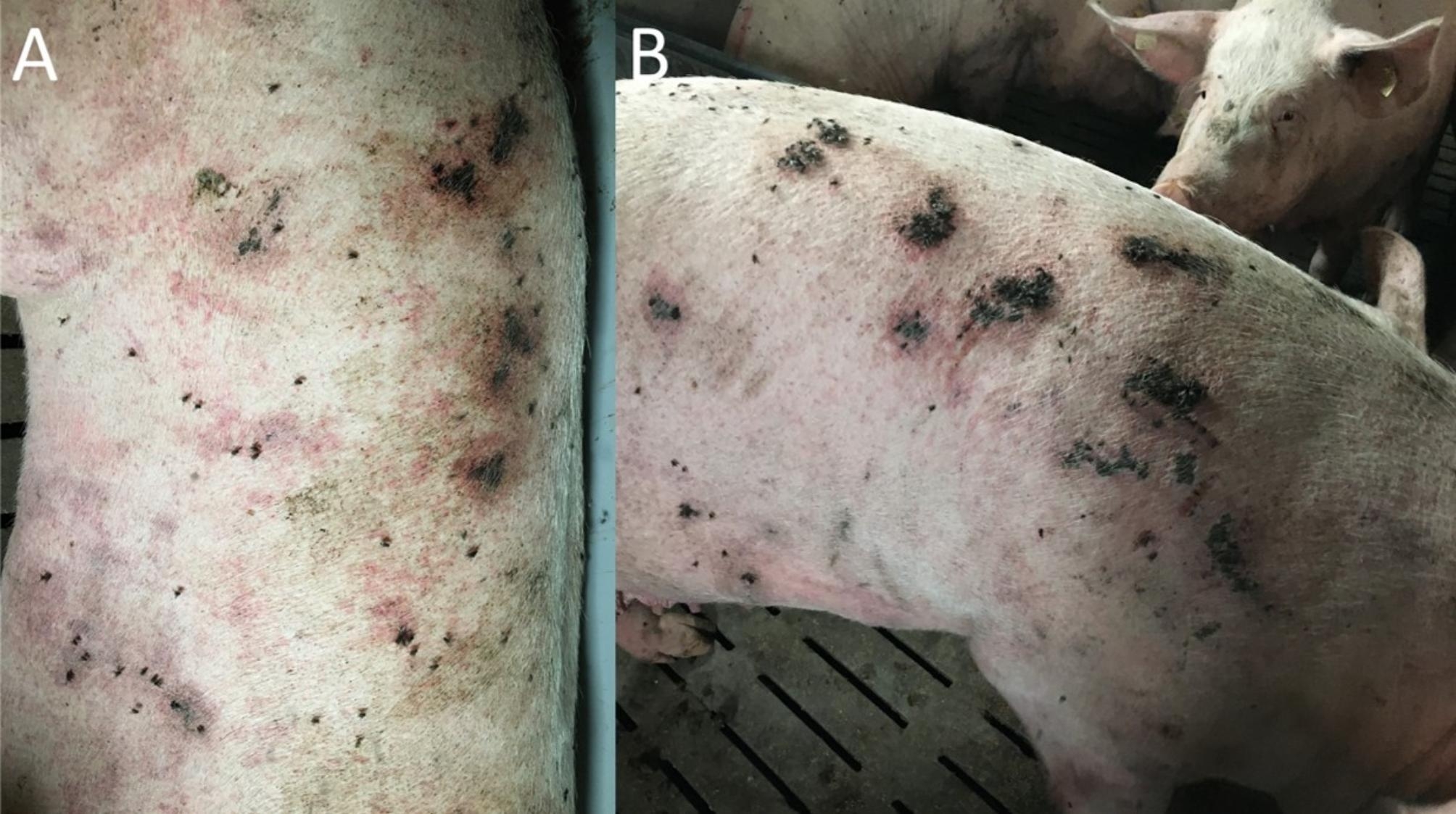
Sows with several characteristic bleeding skin lesions and reddish to purple maculae (**A**) covered with stable flies that were having blood meals (**B**)

**Fig. 2:**
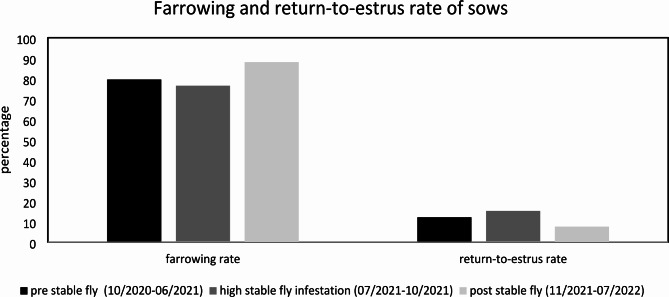
Overview of the reproductive performance of sows before, during and after the outbreak of stable flies in the gestation unit. Recorded data was available to calculate the gestation rate and the return-to-oestrus rate. Sows in gestation were checked by real time ultrasonography

A herd inspection was performed by veterinarians of the University Clinic for Swine. It had to be found out which fly species dominated in the gestation area and if the flies may be related with the skin lesions. All areas of the case farm were inspected starting with the farrowing units and ending at the gestation unit. At the time when the herd inspection was performed, 55 sows and one teaser boar were kept there. 33/55 sows showed moderate to severe skin lesions: bleeding skin parts, bloody crusts and red maculae (Fig. 1A) diffusely distributed over the whole body surface almost always covered by several flies (Fig. 1B). When looking in detail on the flies, 90% of the flies sitting on the skin lesions were individuals of *Stomoxys calcitrans*. The remaining 10% of the insect population consisted of *Musca domestica*, *Drosophila* spp. and species from the family of *Psychodidae*. Stable flies were not only found on the animals, but the majority was found resting on the walls and the ceiling of the gestation unit, a common behaviour of stable flies after having had blood meals (Supplementary File 1). Sows in the gestation area were evaluated as being stressed and showed permanent avoidance behaviour due to fly bites. Sows were lying and resting for short periods of a few minutes only to stand up again and escape from the stable flies. Some sows gave up standing up and escape from flies and stayed in resting position, but still showed reactions such as repelling flies with their legs or cutaneous reflex (Supplementary File 2). Rectal body temperature of bitten sows was not increased, and no further clinical symptoms could be observed. In the other different compartments of the case herd stable flies could be observed as well, but at much lower numbers. Sows in the farrowing unit similarly presented with reddened skin parts and lesions, but these were in remission and in a healing process, as there were only single stable flies present in the farrowing units. Sows in the farrowing units were subjectively evaluated as relaxed compared to the ones in the gestation unit showing low stress levels, as during inspection none of the sows reacted on our presence and either proceeded in resting in lateral recumbency or in suckling their litter. In the nursery unit, single stable flies could be observed on the walls of the different pens. In general, on a semi-quantitative level the infestation level with stable flies in the farrowing and nursery units was evaluated as low (a maximum of one stable fly sitting on one animal) whereas it was high in the gestation area (more than ten stable flies sitting on one animal). Reproduction data of sows, specifically farrowing rate and return-to-oestrus rate was divided into three parts: the time before the massive growth of fly populations in July 2021, the time from July 2021 until the end of October 2021 when the gestation area was heavily overpopulated with flies or stable flies and the time after October 2021 when rigorously insecticides were used to control flies. Mean values showed that during the time of fly overpopulation, the farrowing rate (83.33%) decreased due to an increased return-to-oestrus rate (16.67%). Once the fly population was almost decreased to a zero level after usage of insecticides the farrowing rate increased again (92.10%), and return-to-oestrus rate decreased to an acceptable level of 7.9% (Fig. 2).

**Fig. 3:**
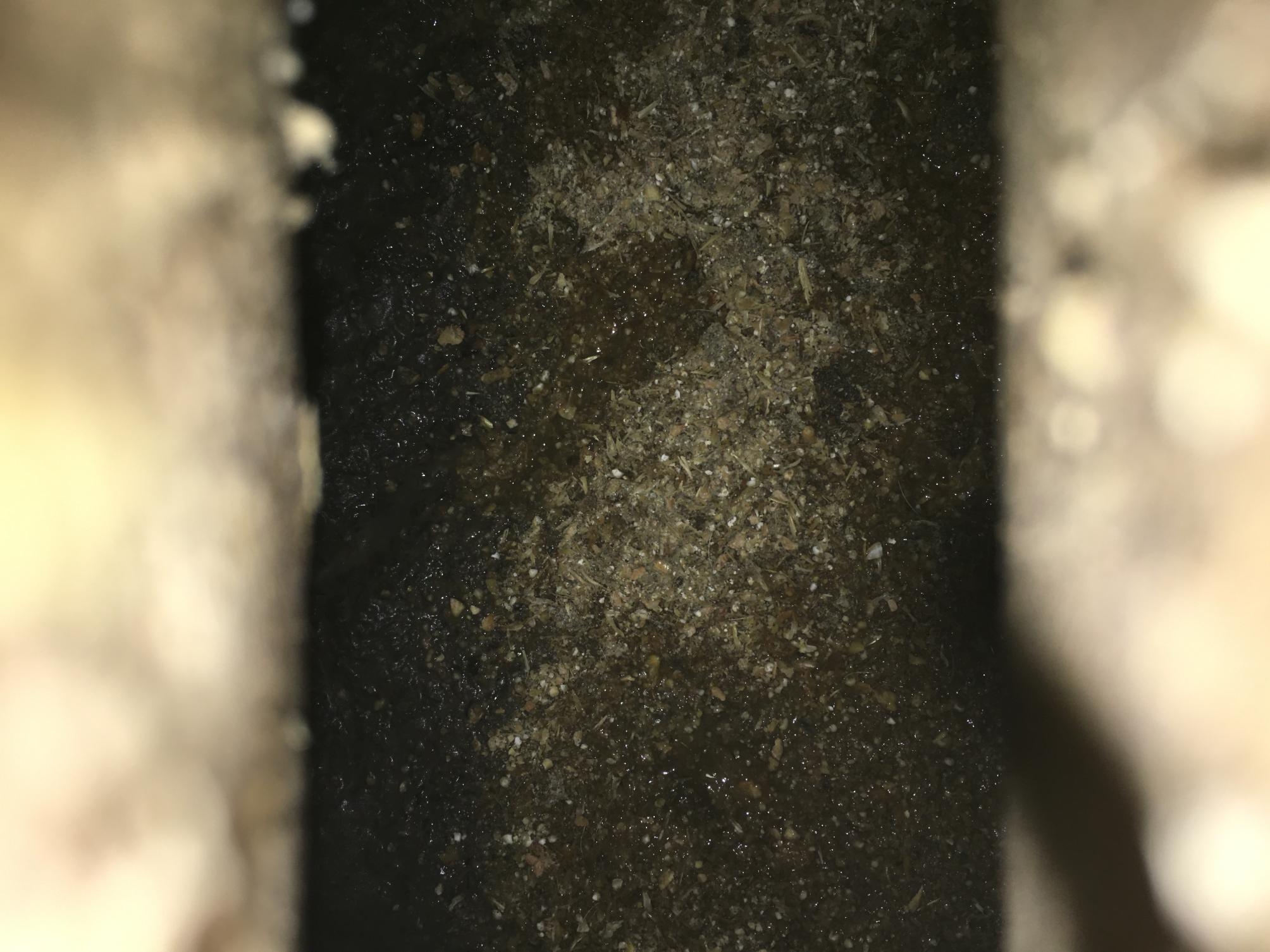
Wasted feed in the slurry channel of the nursery unit starting to decompose and to build a manure cover

To find out risk factors contributing to the huge stable fly population, the whole internal environment of the case herd was inspected with special focus on finding decomposing organic plant material, as this is known to be the main substrate for stable fly maggots to effectively develop [4–6]. In the gestation area and in the farrowing unit no relevant sources of decomposing organic matter could be found. However, a completely different situation was seen in the nursery unit where huge amounts of piglet feed were found below the slatted floors on the slurry starting to build a manure cover (Fig. 3). As the farmer reported of the transfer of liquid manure from the nursery unit to the gestation area to increase liquidity of the slurry and to break the manure cover there, the slurry channel of the gestation area was checked again for spots of decomposing organic matter. In some areas it was possible to find accumulation of decomposing feed on the manure cover in the gestation area together with typically long shaped pupae most probably of stable flies.

### Microbiologic investigations

Swab samples of the skin lesions from four representative sows and stable fly specimen (4 pooled samples containing 10 individuals each) were collected for microbiological analysis. In all four swab samples high grade methicillin-resistant *Staphylococcus* (*S.*) *aureus* (MRSA) and *S. hyicus* (SH) could be found. In three samples high grade of *Streptococcus dysgalactiae* subsp. *equisimilis* (SDSE) and in one high grade *S. microti* was detected. All bacterial species were identified to the species level by matrix-assisted laser desorption ionization-time-of-flight mass spectrometry (MALDI-TOF MS) (Bruker Daltonik, Bremen, Germany) and further characterized by susceptibility testing and in case of MRSA the isolate of the swab of Sow 1 and Sow 4 exemplarily genotyped for all MRSA isolates by *spa*-, *dru-*typing and DNA based microarray [9]. A summary of the results of microbiological investigations can be found in Supplementary File 3. Genes coding for exfoliative toxins of *S. hyicus* were analysed as previously described [10]. MRSA isolates belonged to *spa* type t034, *dru* type dt6j, SCC*mec t*ype V/VT and clonal complex (CC) 398. Genetic resistance matched the phenotypic resistances of the respective isolates. The *mecA, blaZ, tet*(K) and *tet*(M) were observed. Full hybridisation results for MRSA isolates examined in this study can be found in Supplementary File 4. The PCR characterization of *S. hyicus* exfoliative toxin genes revealed that all isolates did not harbour any of examined genes. The preparation of stable flies was as previously described [8]. For selective isolation of *Staphylococcus* sp., 200 µl of suspension containing bacteria from the fly surface were preincubated at 37 °C overnight in buffered peptone water (BPW) (Merck, Germany), wherefrom 200 µL were cultured at 37 °C in trypticase soy broth (TSB) (Becton Dickinson (BD), Heidelberg, Germany) with 6.5% (w/v) NaCl overnight and subsequently streaked onto a BBL CHROMagar MRSA II (Becton Dickinson, Heidelberg, Germany(BD)) and on Columbia CNA Improved II Agar with 5% (v/v) sheep blood (BD). All isolates showing typical staphylococcal colony appearance were identified by MALDI-ToF-MS revealing the presence of *S. hyicus* and *S. simulans* in each of the four stable fly suspensions. The susceptibility testing and the PCR characterization of *S. hyicus* exfoliative genes did not differ from the porcine isolates.

### Recommendations to the farmer and further outcome

Veterinarians from the University Clinic for Swine together with the herd attending veterinarian and the farmer analysed the fly control program of the case farm for weak points that may be improved in future for prevention of any insect overpopulation. A major point for improvement was the time point of starting with insect control. The farmer reported that after cleaning the gestation area in July 2021 it took approximately two weeks until a massive fly population was recognized. Furthermore, the second increase of the fly population occurred at the beginning of October 2021 when liquid manure from the nursery unit was directed in the slurry channel of the gestation area. As plenty of wasted feed was observed on the manure cover of the nursery unit, it was hypothesized that once directed into the slurry channel of the gestation area the decomposing process started and may have established an important source for stable fly reproduction in the gestation area. The farmer was advised to regularly empty slurry channels of all different compartments directly into the slurry tanks to prohibit the transfer of wasted nursery feed. Additionally, he was advised to install fully non-slatted plates around the feeding troughs to reduce the spill over of feed directly into the slurry channel.

Since the high population density of stable flies in the gestation unit needed fast intervention to improve animal welfare and health of sows quickly, we recommended the use of adulticides together with larvicides to quickly and sustainably reduce the relative abundance of the stable flies close to zero.

We recommended to the farmer, whenever the relative abundance of stable flies increases in any of the different compartments of the case herd, control measures have to be applied immediately to stop further reproduction and spread within the farm. Waiting too long with starting fly control measures can have substantial impact on the reproduction rate of the stable fly population, as one female may produce a maximum of 820 eggs [11]. Control measures referred to removal and reduction of reproduction niches of stable flies to break the development of maggots [2].

Regarding the skin lesions of sows, we recommended local treatment using zinc or silver sprays. In any case constitution of sows suffering from skin lesions should get worse, antimicrobial treatment of skin lesions with a thiamphenicol spray together with nonsteroidal antiphlogistic drugs was recommended.

According to the farmer, skin wounds healed without any therapeutic intervention once the adult and larval stable fly population was reduced and controlled. The farmer also reported that it took approximately four weeks until skin lesions healed, and reproductive performance reached again acceptable levels. One year after the massive outbreak of stable flies in the gestation unit, just single specimens of *Stomoxys calcitrans* were reported by the farmer, assuming that controlling reproduction of stable flies was successful. Since October 2021 no further massive occurrence of stable flies was observed in the case herd.

## Discussion and conclusion

The biology and reproduction of stable flies was studied in history quite well, but controlling this nuisance pest insect still seems anything but trivial compared to house flies [2, 5, 12]. First of all, there is the different feeding behaviour of stable flies as blood suckers which make it difficult to control adult flies using insecticidal baits such as granulated formulations, because stable flies are not attracted by them enough. Cook reviewed the different strategies for controlling stable flies and pointed out the importance of minimising pesticide usage and focusing on alternative control measures such as control of fly reproduction or biological control [13]. Female stable flies especially are attracted by decomposing organic material, mainly of plant origin, such as different sorts of straw mixed with manure [3, 4, 6, 14]. In pig farms decomposing organic material can be found especially around the feeding troughs and in farms using deep straw bedding. The case herd used slatted flooring systems, consequently during the herd inspection it was crucial to find other sources of decomposing organic matter. In the nursery unit plenty of wasted feed that fell directly on the manure cover through the slatted floor could be identified as one major source of organic material. However, the farmer of the case herd described the first massive fly occurrence after he had cleaned and washed the gestation unit and the second one shortly after he directed the liquid manure from the nursery unit into the slurry channel of the gestation unit. Since we were not able to retrospectively determine the predominant fly species at the first fly population increase, we just can speculate. But since the farmer did not observe any skin lesions on sows similar to the ones observed in October 2021, we assume that house flies may have been the predominant fly species and stable flies just occurred at low abundance. This situation changed totally, as in October 2021 sows in the gestation unit developed moderate to severe bleeding skin lesions and bloody crusts. During the herd inspection the majority of flies were determined as *Stomoxys calcitrans* with an estimated relative abundance of 90%. Sows’ behaviour was affected due to partly massive stable fly attacks on the whole body surface with a focus on the already existing bleeding skin lesions. Predominant behavioural changes were defensive behaviour, spontaneous flight responses to escape from stable fly bites, increased scratching activity and restlessness. These behavioural changes were already described in literature but mostly were true for cattle as hardly any literature on the impact of stable flies on the behaviour of pigs exist [1, 2, 12, 13]. Economic effects were mostly investigated in cattle and dairy farms regarding weight gain and milk production [15–17]. Based on the recorded reproductive performance data of the case herd the return-to-oestrus rate increased from 12.17 to 15.31% and gestation rate decreased from 79.57 to 76.53% in the time from July 2021 till the end of October 2021 when fly population overgrew. However, once insecticides (adulticides and larvicides combined) to control the stable fly population were used, the relative abundance of the insect population in the gestation area reached a level close to zero and reproductive performance parameters improved to a level exceeding the time prior to a stable fly overpopulation in the gestation unit with flies: 88.07% gestation rate and 7.58% return-to-oestrus rate (Fig. 3). Most probably the reduced reproductive performance of sows in the case herd was caused by stress due to the massive attacks of sows by stable flies.

A high relative abundance of stable flies in closed buildings is not only a health issue, but rather an animal welfare issue, as the bitten sows could not escape to an environment without stable flies consequently resulting in an increased stress level. At a certain time point sows stop their repellent behaviour and just accept being bitten by stable flies as is shown in Supplementary File 2. This phenomenon is already known from historic reports of unusual outbreaks of *Stomoxys calcitrans* in northern Texas when livestock showed anxiety due to stable fly attacks and at a certain stage gave up fighting [3]. While house flies mostly are nuisance insects with less direct harming effects, stable flies have important impact on animal welfare and health due to painful bites that additionally may transmit pathogens and cause blood loss [1]. Stable flies ingest on an average 11–15 µl blood per meal and female stable flies feed at least three to four times until they deposit eggs [3, 18]. Blood loss in sows was assumed to be substantial. Therefore, this case points out the importance of proper insect control in food animal production to increase health and welfare of livestock.

As already recommended to the farmer to control reproduction of the stable fly in certain cases insecticides may be the first choice to reduce the relative abundance and consequently to increase animal health and welfare. Once stable fly population was controlled, preventive measures such as regular direct emptying of slurry channels into the slurry tank, preventing feed wasting and removing reproduction sites of stable fly maggots were enough to control fly population in the case herd. However, sustainable control of insect population in farm animal production should be implemented via an integrated pest management [13, 19]. Such concepts have already been published for ruminant operations and the authors concluded that control of pest development sites and monitoring of harmful pest species were seriously achievable goals for the near future [19]. Hence, this may be also true for porcine operations. One major problem in controlling insects in pig production systems is the fact that most often control measures were not applied at the right time point, as it was the case in the reported farm. Integrated insect management must be done continuously and in time to reach the main goal: minimising pesticide usage through monitoring, prevention and the use of alternatives to pesticides such as parasitoids and entomopathogenic organisms [13, 19, 20]. Pesticides were used over decades in insect control, but there is a rising number of reports of strains of stable and house flies resistant to pesticides [20–24]. Farmers and herd attending veterinarians must have the necessary awareness of the harming effects not only of stable flies, but rather of all insects in pig farms. Further research focusing on the role and impact of stable flies in pig production have to be performed with special focus on the economy, animal welfare and animal health in the context of one health. As prevalence studies on *Stomoxys calcitrans* in Austrian pig production systems are lacking, we just can speculate about the economic losses due to the stable fly.

From a microbiological point of view, stable flies can act as important living vectors of bacteria and viruses [1, 25–29]. In a previous study bacterial communities on stable flies have been investigated resulting in the detection of 69 different microorganisms including also characteristic skin microbiota, such as *Mammiliicoccus sciuri* (formerly: *S. sciuri*) and *S. epidermidis* [8]. Swab samples taken from bleeding skin lesions from four representative sows revealed MRSA, *S. hyicus* and SDSE. All three bacteria are known to colonize skin wounds and to produce chronic skin diseases such as exudative epidermitis under certain circumstances [30, 31]. MRSA isolates belonging to CC398 are the most common livestock-associated lineage of (MRSA) in Western Europe [32]. CC398-MRSA-V/VT is mainly associated with pigs, but also with cattle, humans and companion animals [32, 33]. CC398 is known to be associated with different diseases in pigs [33], and can cause serious infections and outbreaks in humans [34, 35]. Thus, the results of the present study strongly support strengthening of hygiene measures. The same applies in case of *S. hyicus*, a well-known porcine pathogen [36]. Even though S. *hyicus* is rarely associated with infections in humans [37], its potential for causing infections in pigs and humans should also be considered once hygiene measures are applied.

Controlling insects in large animal production should be a key element in future management concepts to minimize risk of any harm to animals and humans. Synantropic flies such as stable flies and house flies are living vectors of different bacterial agents which may be transferred via short distances of approximately three kilometres to other animal stocks or humans [38–40]. Therefore, stable flies play an important role in the one-health context due to their potential transmission of bacteria, which carry resistance genes against antimicrobials. SH detected in swab samples from the skin lesions of sows could also be isolated from stable flies after washing in sterile water. Hence, stable flies can serve at least as vehicles for SH but most probably also for MRSA being transferred from carrier pigs to other hosts. Taking into account that stable flies and most probably also house flies of the case farm were at least carrying SH on their body surface, this necessitates a proper control of the fly population to prohibit further spread of bacteria resistant to antimicrobials.

In conclusion, stable flies in piglet producing farms can have important impact on animal health and animal welfare. Massive overpopulation with *Stomoxys calcitrans* can lead to severe affection of sows’ skin resulting in bleeding skin lesions. Increased stress levels due to steady repellent behaviour of sows to fight against stable fly bites and due to inability to leave to areas that are free of stable flies were assumed to be the reason for reduced reproductive performance. Overall, *Stomoxys calcitrans* should be recognized as important harmful pest insects in swine production.

### Electronic supplementary material

Below is the link to the electronic supplementary material.

Supplementary Material 1: Movie demonstrating the massive amount of stable flies resting on the ceiling of the gestation unit.

Supplementary Material 2: Movie of one separated sow that resigned fighting against stable flies, but still showed reactions such as cutaneous reflexus and attempts to remove stable flies with its legs.

Supplementary File 3: Summary of the main molecular characterization, antimicrobial resistance and toxins profile of MRSA, SDSE and other staphylococci investigated.

Supplementary Material 4: Full hybridisation results for MRSA isolates examined.

## Data Availability

All data generated and analysed during this case report are included in this article or are available as supplementary files.
