# Supplementary material for: Bleeding skin lesions in gestating sows of a piglet producing farm in Austria
Source: Porcine Health Manag. 2023 Nov 14;9:52. doi: 10.1186/s40813-023-00348-4 (PMC10647148; doi:10.1186/s40813-023-00348-4)
Supplement: Supplementary file 3 — Summary of the main molecular characterization, antimicrobial resistance and toxins profile of MRSA, SDSE and other staphylococci investigated. [file 40813_2023_348_MOESM3_ESM.docx]

| Supplementary File 3: Summary of the main molecular characterization, antimicrobial resistance and toxins profile of MRSA, SDSE and other staphylococci investigated   \| Isolates \| Species \| CC^1^ \| *spa* \| *dru* \| Antimicrobial resistance profile \| \| \| --- \| --- \| --- \| --- \| --- \| --- \| --- \| \| Phenotype \| Genes detected \| \| Sow 1, 4 \| MRSA \| CC398 \| t034 \| dt6j \| β-lactams, fluoroquinolones, macrolides, lincosamide, ERY, TET \| *mecA, blaZ, blaI, blaR, erm*(A)*, tet*(K)*, tet*(M) \| \| Sow 1, 2, 4 \| SH \| n.a. \| n.a. \| n.a. \| fully susceptible \| n.a. \| \| Sow 3 \| SH \| n.a. \| n.a \| n.a. \| PEN \| n.a. \| \| Sow 2, 3, 4 \| SDSE \| n.a. \| n.a. \| n.a. \| TET, CLI, STX \| n.a. \| \| Sow 4 \| SM \| n.a. \| n.a. \| n.a. \| TET \| n.a. \| \| Pooled stable flies \| SH \| n.a. \| n.a. \| n.a. \| PEN \| n.a. \|  \| Isolates \| *cap* gene (*cap* 5) \| Hemolysins \| Leukocidins (Luk) \| Biofilm-associated genes \| Adhesion factors \| Exfoliative toxins \| Enterotoxins and enterotoxin-like genes \| \| --- \| --- \| --- \| --- \| --- \| --- \| --- \| --- \| \| \| Sow 1, 4 \| POS \| *hla, hlb, hld, hlgA* \| *lukF, lukS, lukX, lukY* \| *icaA, icaC, icaD* \| *clfA, clfB, cna, fnbA, fnbB* \| NEG \| NEG \| \| Sow 1, 2, 4 \| n.a. \| n.a. \| n.a. \| n.a. \| n.a. \| NEG \| n.a. \| \| Sow 3 \| n.a. \| n.a. \| n.a. \| n.a. \| n.a. \| NEG \| n.a. \| \| Sow 2, 3, 4 \| n.a. \| n.a. \| n.a. \| n.a. \| n.a. \| n.a. \| n.a. \| \| Sow 4 \| n.a. \| n.a. \| n.a. \| n.a. \| n.a. \| n.a. \| n.a. \| \| Pooled stable flies \| n.a. \| n.a. \| n.a. \| n.a. \| n.a. \| NEG \| n.a. \| |
| --- | --- | --- | --- | --- | --- | --- | --- | --- | --- | --- | --- | --- | --- | --- | --- | --- | --- | --- | --- | --- | --- | --- | --- | --- | --- | --- | --- | --- | --- | --- | --- | --- | --- | --- | --- | --- | --- | --- | --- | --- | --- | --- | --- | --- | --- | --- | --- | --- | --- | --- | --- | --- | --- | --- | --- | --- | --- | --- | --- | --- | --- | --- | --- | --- | --- | --- | --- | --- | --- | --- | --- | --- | --- | --- | --- | --- | --- | --- | --- | --- | --- | --- | --- | --- | --- | --- | --- | --- | --- | --- | --- | --- | --- | --- | --- | --- | --- | --- | --- | --- | --- | --- | --- | --- | --- | --- | --- |

Legend: MRSA = methicillin-resistant *Staphylococcus aureus*; SH = *Staphylococcus hyicus;* SDSE = *Streptococcus dysgalactiae* subsp*. equisimilis; SM = Staphylococcus microti;* CC = clonal complex; ERY = erythromycin; PEN = penicillin; STX = sulfonamide/trimethoprim; TET = tetracycline; NEG = negative, POS = positive, n.a. = not assessed;.
